# Ameliorating Effects of Ethanol Extract of *Fructus mume* on Scopolamine-Induced Memory Impairment in Mice

**DOI:** 10.1155/2015/102734

**Published:** 2015-02-01

**Authors:** Min-Soo Kim, Won Kyung Jeon, Kye Wan Lee, Yu Hwa Park, Jung-Soo Han

**Affiliations:** ^1^Department of Biological Sciences, Konkuk University, Seoul 143-701, Republic of Korea; ^2^Herbal Medicine Research Division, Korea Institute of Oriental Medicine, Daejeon 305-811, Republic of Korea; ^3^Dongkook Pharm. Co., Ltd., R&D Center, 147 Gwanggyo-ro, Yeongtong-gu, Suwon-si, Gyeonggi-do 443-270, Republic of Korea

## Abstract

We previously reported that *Fructus mume* (*F. mume*) extract shows protective effects on memory impairments and anti-inflammatory effects induced by chronic cerebral hypoperfusion. Neurodegeneration of basal cholinergic neurons is also observed in the brain with chronic cerebral hypoperfusion. Therefore, the present study was conducted to examine whether *F. mume* extracts enhance cognitive function via the action of cholinergic neuron using a scopolamine-induced animal model of memory impairments. *F. mume* (50, 100, or 200 mg/kg) was administered to C57BL/6 mice for 14 days (days 1–14) and memory impairment was induced by scopolamine (1 mg/kg), a muscarinic receptor antagonist for 7 days (days 8–14). Spatial memory was assessed using Morris water maze and hippocampal level of acetylcholinesterase (AChE) and choline acetyltransferase (ChAT) was examined by ELISA and immunoblotting. Mice that received scopolamine alone showed impairments in acquisition and retention in Morris water maze task and increased activity of AChE in the hippocampus. Mice that received *F. mume* and scopolamine showed no scopolamine-induced memory impairment and increased activity of AChE. In addition, treatments of *F. mume* increased ChAT expression in the hippocampus. These results indicated that *F. mume* might enhance cognitive function via action of cholinergic neurons.

## 1. Introduction

*Fructus mume* extract, which is used in the treatment of chronic diarrhea and lingering dysentery in Asian countries, has been reported to be effective against colitis in an animal model [1]. The efficacy and therapeutic mechanisms of* F. mume* on macrophage-mediated inflammation were also demonstrated by the finding that* F. mume* treatment inhibited proinflammatory mediators in lipopolysaccharide-stimulated RAW 264.7 cells [2]. Recently, the effects of* F. mume *oncognitive function were examined using a bilateral common carotid artery occlusion (BCCAo) animal model [3], which has been used to study the contribution of chronic cerebral hypoperfusion to the development of cognitive decline in vascular dementia (VaD) and to develop therapeutic treatments [4–6]. Rats subjected to chronic cerebral hypoperfusion with daily administration of* F. mume* showed reduced impairment of spatial memory relative to the BCCAo rats. Further, administration of* F. mume *mitigated BCCAo-induced microglial activation [3].

It has also been reported that animals with chronic BCCAo showed alterations of cholinergic markers in the hippocampus [7–9]. Neurodegeneration of basal forebrain cholinergic neurons (BFCNs) has been observed in patients with VaD and Alzheimer's disease (AD) associated with loss of cognitive function. Hence, there is a possibility that the preservation of cognitive function could be due to a greater number of intact BFCNs following* F. mume *treatment in rats with BCCAo.

The BFCNs play a key role in the processes of cognitive function, including memory [10, 11]. For example, BFCN lesions, which decrease acetylcholine (Ach) release into the synaptic cleft, result in learning and memory dysfunction [12] or loss of attention [13]. The duration of action of the neurotransmitter ACh depends on the activity of acetylcholinesterase (AChE), which hydrolyzes ACh after its release [14]. Therefore, inhibition of AChE activity is the main therapeutic approach for Alzheimer's disease [15]. Choline acetyltransferase (ChAT), an enzyme that synthesizes Ach, is a specific marker of cholinergic neurons [16].

Scopolamine, which has been used for inducing memory impairment for the screening of antiamnesic drugs, is a muscarinic ACh receptor (mAChR) antagonist [17–19]. The hippocampus, as part of a critical system for the formation of stable memories [20, 21], is particularly vulnerable to scopolamine treatment [22]. Scopolamine treatment increases AChE levels in the hippocampus [23]. Therefore, in order to examine the effects of* F. mume* on cognitive function and the cholinergic system, the present experiment was conducted to evaluate learning and memory retention using the Morris water maze task and to measure changes in the cholinergic markers (AChE and ChAT) in scopolamine-treated C57BL/6 mice that received* F. mume.* In addition, based on the recent study demonstrating that the ethanol extract of* F. mume* stimulated glucose uptake more than its water extract in C2C12 myotubes [24], we used the ethanol extract of* F. mume* in the present study.

## 2. Materials and Methods

### 2.1. Animals

Fifty-seven male C57BL/6 mice were used in the experiment (8 weeks old; Charles River Co., Gapyeong, South Korea). The mice were housed in a vivarium at Konkuk University for 2 weeks at the beginning of the experiment under controlled temperature (22 ± 1°C) and humidity (50 ± 10%) on a 12 h light/dark cycle (lights on at 0800 h). Food and water were provided* ad libitum* to all animals throughout the experiment. The Institutional Animal Care and Use Committee of Konkuk University approved all protocols described in the report. All experimental treatments and behavioral testing took place during the light phase.

### 2.2. Preparation of* F. mume* Extracts and Administration

*F. mume*, the dried unripe fruit of* Prunus mume*, was purchased from a commercial supplier (Dongwoodang Pharmaceutical Co., Yeongcheon, Korea) in 2014. It was identified by the Herbal Medicine Research Division of the Korea Institute of Oriental Medicine (KIOM) and deposited at the R&D Center of the Dongkook Parmaceutical Co., Korea. Dried* F. mume* was pulverized and extracted with 70% ethanol (25 kg/200 L) for 3 h at 60°C in an ultrasound-assisted extractor (OM30-EP; Sonimedi, Korea). All extracts were concentrated under vacuum using a rotary evaporator after filtration and were then dried for 48 h at 40°C by using an extract vacuum drier (Exdryer, Sonimedi, Korea) to yield a powder extract (4.0 kg, 16% yield). The powder extract was suspended in sterilized distilled water at the appropriate concentrations. A high-performance liquid chromatography (HPLC) assay was performed with benzyl-*O*-*β*-d-glucopyranoside as a standard maker for quality control of the composition of the* F. mume *extract in each experiment [25]. HPLC was performed using a Waters 2695 alliance, a 2487 dual absorbance detector, and a Capcell pak UG120, C18 (5 *μ*m, 4.6 × 250 mm i.d.). The mobile phase comprised water (A) and acetonitrile (B) with a stepwise gradient elution mixture: 0 min, 5% B; 8 min, 10% B; 15 min, 30% B; 30 min, 50% B; 31 min, 5% B; 38 min, 5% B. The flow rate was 1.0 mL/min.

The* F. mume *extract was filtered on membrane filters with a 0.45 mm pore size (Millipore) and a 10 *μ*L injection volume. Benzyl-*O*-*β*-d-glucopyranoside was detected at a wavelength of 210 nm. The crude extract was analyzed in triplicate, and the benzyl-*O*-*β*-d-glucopyranoside content was found to be about 0.182%.

**Figure 1:**
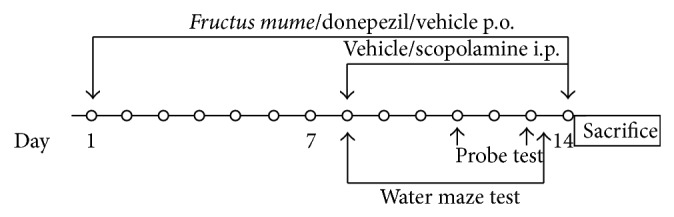
Experimental procedure. After 5-day adaption period, C57BL/6 male mice received vehicle (saline),* Fructus mume* (50, 100, or 200 mg/kg, p.o.), or donepezil (5 mg/kg) for 14 days. On day 8, vehicle (saline) or scopolamine (1 mg/kg, i.p.) was also administered for 7 days, and the Morris water maze task was conducted to assess cognitive function during this period (days 8–13). On day 14, each mouse was sacrificed, and the hippocampus was removed for the analysis of acetylcholinesterase activity and choline acetyltransferase expression.

The mice used in the experiment were segregated into six groups: (1) vehicle + vehicle; (2) vehicle + scopolamine; (3)* F. mume* (50 mg/kg) + scopolamine; (4)* F. mume* (100 mg/kg) + scopolamine; (5)* F. mume* (200 mg/kg) + scopolamine; and (6) donepezil (5 mg/kg) + scopolamine.* F. mume* was dissolved in saline and administered daily by oral gavage (p.o.) on days 1–7 for adaptation to oral administration and metabolism, prior to training for the Morris water maze test, and continued on days 8–14, during which the mice underwent the Morris water maze test. Scopolamine (1 mg/kg per day) was dissolved in saline and administered by intraperitoneal (i.p.) injection on days 8–14. On days 8–14,* F. mume* and scopolamine were administered 60 min and 30 min before the trial, respectively. The group 1 (vehicle + vehicle) was used as control group; mice were administered with oral saline administration on days 1–14 and additional saline intraperitoneal injection on days 8–14. On day 14, the mice were decapitated 30 min after injection of scopolamine or vehicle (Figure 1). The hippocampus was removed, dissected on ice, and frozen at −80°C until analysis.

### 2.3. Morris Water Maze Task

The water maze consisted of a circular tank (1.83 m diameter and 0.58 m height) with an escape platform (20 cm diameter) centered in one of the four maze quadrants. The water (27°C) was made opaque with nontoxic white paint. The hidden platform was located 0.5 cm beneath the surface for place training. The maze was surrounded by white curtains, on which black felt patterns were affixed to provide distal visual (spatial) cues. Mice received 4 trials/day (10 min intertrial interval, maximum trial duration of 60 s, with 20 s on the platform at the end of each trial), with each trial beginning at one of four equidistantly located positions at the perimeter of the maze (days 8–13). The location of the platform remained constant across all training trials. Mice were placed into the water facing the wall and were allowed to swim for a maximum of 60 s. The trial ended when a mouse climbed onto the available platform or after the 60 s interval had elapsed. If a mouse did not locate the platform during a trial, it was placed on the platform by the experimenter. Mice were left on the platform for 20 s and then moved to a holding cage for a 10 min intertrial interval. During the training (acquisition) trials, search errors that are described in detail elsewhere [26] were used to assess the performance accuracy of spatial learning in the water maze. During each trial, the distance of the mice from the escape platform was sampled 10 times per second, and these values were averaged in 1 s bins. The cumulative search error was then calculated as the summed 1 s averages of the proximity measures corrected for the particular start location in each trial. On day 11 and 13, 30 min after the last trial, the platform was removed from the tank, and the mice underwent a spatial probe trial in which they were given 60 s to search for the removed platform. The time spent in the target quadrant was measured. A camera located above the center of the maze relayed images to a videocassette recorder. Data from the water maze trials were analyzed using human visual system Image Software (HVS Image, Hampton, UK).

### 2.4. AChE Activity Analysis

An enzyme-linked immunosorbent assay (ELISA) for measuring AChE levels in the hippocampus (The Amplex Red ACh/AChE assay kit, Molecular probes, Grand Island, NY, USA) was used to determine AChE activity. Working solutions of 400 *μ*M Amplex Red reagent containing 2 U/mL horseradish peroxidase and 0.2 U/mL choline oxidase were prepared from stock solutions. To measure the effect of* F. mume* on AChE activity in tissues, 100 *μ*M ACh was added to measure the AChE activity. The reaction began when 100 *μ*L of the working solution was added to microplate wells containing the samples. The fluorescence emitted by the individual samples was detected using a Fluorescence microplate reader (Molecular Devices, Sunnyvale, CA, USA) at an excitation wavelength of 560 nm and emission wavelength of 590 nm.

### 2.5. Western Blot Analysis

Tissues were homogenized in radioimmunoprecipitation assay buffer (Cell Signaling Technology, Beverly, MA, USA). Homogenates were centrifuged at 14,000 ×g for 30 min. Supernatants were collected, and protein concentrations were determined using Bradford's methods. The protein was separated using 10% sodium dodecyl sulfate polyacrylamide gel electrophoresis and electrophoretically transferred to a polyvinylidene fluoride membrane (Millipore, Billerica, MA, USA). The membrane was blocked in 5% fat-free dry milk and then incubated with primary antibodies against ChAT (1 : 1000, Millipore) and *β*-actin (1 : 5000, Sigma). After incubation with horseradish peroxidase conjugated secondary antibodies, protein bands were detected using an enhanced chemiluminescence detection kit (GE Healthcare, St. Giles, UK). The protein bands were scanned and measured using the Image Gauge program (GE Healthcare).

### 2.6. Statistical Analysis

One-way analysis of variance (ANOVA) and one-way repeated ANOVA were conducted to assess the effects of the* F. mume *extract on the changes in hippocampal AChE and ChAT levels and the impairment of spatial memory induced by scopolamine. Post hoc analyses (Least Significant Difference test) were subsequently conducted to determine the effects of* F. mume* treatment. *P* values of less than 0.05 were considered significant, unless otherwise specified. Data were expressed as mean ± standard error of the mean (SEM).

## 3. Results

### 3.1. *F. mume* Alleviated Scopolamine-Induced Memory Impairment

**Figure 2:**
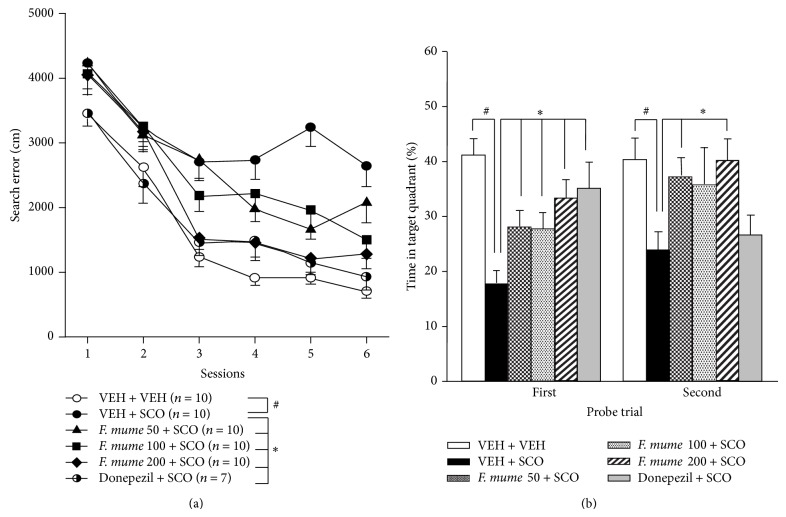
Effect of* Fructus mume* on scopolamine-induced memory impairment. (a) Acquisition of spatial memory in the Morris water maze task. Vehicle control mice (VEH + VEH) were proficient in locating a hidden platform, but scopolamine-treated mice (VEH + SCO) were not (#). Scopolamine-treated mice that also received* Fructus mume (F. mume) *or donepezil showed better performances than those treated with scopolamine alone (∗). (b) Retention of spatial memory. On days 11 and 14, the probe trials were conducted 24 h after the training session. In both probe trials, vehicle control mice spent significantly more time in the target quadrant than the scopolamine-treated mice (#). In both probe trials, scopolamine-treated mice that also received* F. mume *showed better retention of spatial memory than those treated with scopolamine alone (∗). Donepezil showed significant effects on improving retention of spatial memory at the first probe trial only (∗). Data are expressed as mean ± standard error of the mean (SEM).

The Morris water maze test was used to measure the cognitive function of mice treated with* F. mume* or scopolamine. Two-way ANOVA on the mean search error (i.e., mean distance from the escape platform during the search) revealed significant between-group effects (vehicle control, scopolamine,* F. mume *+ scopolamine groups, and donepezil+ scopolamine group) (*F*(5,51) = 25.21, *P* < 0.0001) and training effects (*F*(5,255) = 99.64, *P* < 0.0001). A significant interaction effect between treatment and training was observed (*F*(25,255) = 2.00, *P* = 0.004). As shown in Figure 2(a), the control mice showed improved search error during the training sessions; however, scopolamine-treated mice showed little improvement over the course of training. Post hoc analyses confirmed that the mean search error of the control mice was significantly lower than that of the scopolamine-treated mice (*P* < 0.0001). However, the scopolamine-treated mice that received* F. mume* (50, 100, or 200 mg/kg per day) performed significantly better than those that received scopolamine alone (*P* < 0.05) (Figure 2(a)). Furthermore, scopolamine-treated mice that received donepezil performed significantly better than those that received scopolamine alone (*P* < 0.05) (Figure 2(a)). However, no significant differences were found in swimming speed (cm/s) among any of the groups (*F*(5,51) = 0.329, *P* = 0.893; (1) vehicle + vehicle: 18.7 ± 0.79; (2) vehicle + scopolamine: 17.0 ± 0.83; (3)* F. mume* (50 mg/kg) + scopolamine: 16.5 ± 0.28; (4)* F. mume* (100 mg/kg) + scopolamine: 16.1 ± 0.84; (5)* F. mume* (200 mg/kg) + scopolamine: 16.6 ± 0.93; (6) donepezil (5 mg/kg) + scopolamine: 17.6 ± 1.10), which means that locomotor function in mice was not affected by scopolamine or drug administration.

On day 11 (24 h after the fourth training session), the mice underwent the spatial probe test to determine whether they remembered the platform location. One-way ANOVA revealed significant between-group effects (vehicle control, scopolamine,* F. mume* + scopolamine groups, and donepezil + scopolamine group) (*F*(5,51) = 6.51, *P* < 0.0001). Post hoc analyses were conducted to assess between-group differences. Similar to the results of the acquisition tests, the mean time in the target quadrant (i.e., percentage of time spent in the quadrant containing the target platform) was significantly lower for the scopolamine-treated mice than the vehicle control mice (*P* < 0.05, Figure 2(b)). However, scopolamine-treated mice that received* F. mume* (50, 100, or 200 mg/kg) spent significantly more time in the target quadrant than those that received scopolamine alone (*P* < 0.05, Figure 2(b)). The scopolamine-treated mice also receiving donepezil spent significantly more time in the target quadrant than those treated with scopolamine alone (*P* < 0.05, Figure 2(b)). On day 14 (24 h after the last training session), the mice underwent another spatial probe test. One-way ANOVA revealed significant between-group effects (vehicle control, scopolamine,* F. mume* + scopolamine groups, and donepezil + scopolamine group;* F*(5,51) = 2.58, *P* < 0.05). Subsequent post hoc analyses revealed results similar to those of the first probe, with significant differences between the vehicle-treated and scopolamine-treated mice. Furthermore, scopolamine-treated mice that received* F. mume *(50, 100, or 200 mg/kg) showed significantly better performances compared with those treated with scopolamine alone (*P* < 0.05, Figure 2(b)). But there was no difference between scopolamine-treated mice and scopolamine-treated mice with donepezil treatment.

### 3.2. *F. mume* Attenuated the Scopolamine-Induced Increase of AChE Activity in the Hippocampus

**Figure 3:**
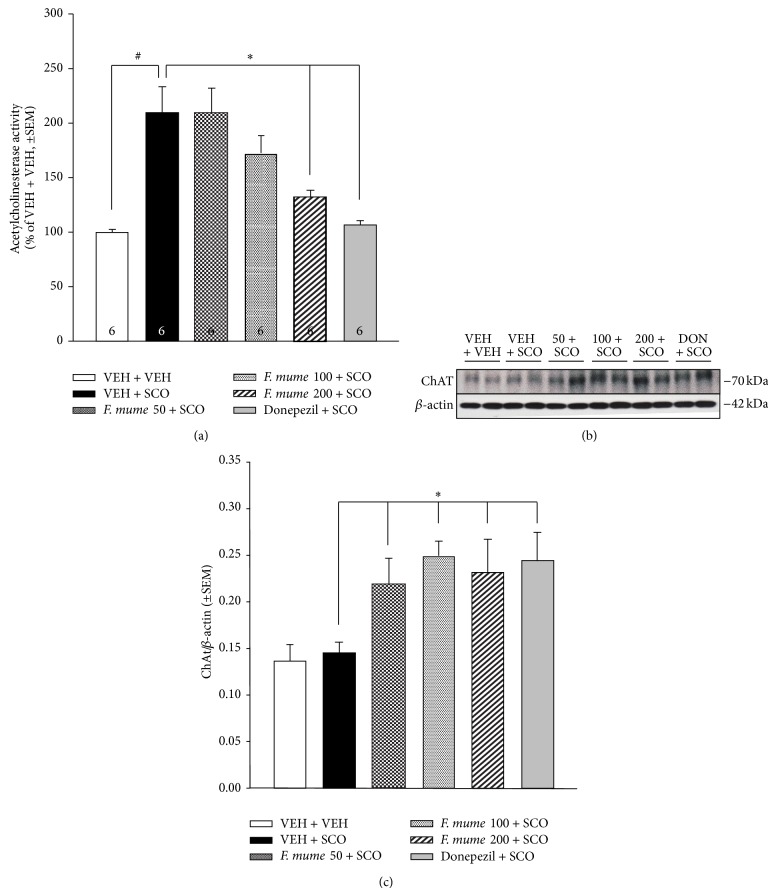
Effect of* Fructus mume* on hippocampal cholinergic markers in scopolamine-treated mice. (a) Hippocampal acetylcholinesterase (AChE) levels. Scopolamine-treated mice (VEH + SCO) showed higher AChE levels in the hippocampus than vehicle control mice (VEH + VEH, #). Scopolamine-treated mice that also received* Fructus mume (F. mume, *200 mg/kg) or donepezil showed decreased levels of hippocampal AChE compared to mice treated with scopolamine alone (∗). (b) Representative western blot of choline acetyltransferase (ChAT). (c) Hippocampal ChAT levels.* F. mume* or donepezil treatment increased hippocampal ChAT levels (∗). The number in the bar graph indicates the *n* size per group. Data are expressed as mean ± standard error of the mean (SEM).

AChE levels in the hippocampus were measured to establish if* F. mume* treatment had an effect on scopolamine-increased AChE levels. One-way ANOVA revealed significant between-group effects (vehicle control, scopolamine,* F. mume* + scopolamine groups, and donepezil + scopolamine group) (*F*(5,30) = 10.37, *P* < 0.0001). According to subsequent post hoc analyses, compared to those of vehicle control mice, hippocampal AChE levels in the scopolamine-treated mice were significantly increased (Figure 3(a)). However, compared with mice treated with scopolamine alone, those that also received* F. mume* treatment (200 mg/kg) showed a significant reduction in hippocampal AChE levels (*P* < 0.05, Figure 3(a)). This reduction was also observed in the scopolamine-treated mice that also received donepezil (*P* < 0.05, Figure 3(a)).

### 3.3. *F. mume* Increased Hippocampal ChAT Expression

Western blot analysis was used to measure hippocampal ChAT levels to establish if* F. mume *treatment influenced hippocampal ChAT expression. ANOVA revealed significant between-group effects (vehicle control, scopolamine,* F. mume* + scopolamine groups, and donepezil + scopolamine group) (*F*(5,30) = 4.07, *P* = 0.006). Subsequent post hoc analyses revealed that hippocampal ChAT levels did not differ significantly between the scopolamine-treated mice and vehicle control mice (Figures 3(b) and 3(c)), which is consistent with an earlier report [23]. However,* F. mume *treatments at all concentrations used in the experiment and donepezil treatment increased hippocampal ChAT expression levels (*P* < 0.05).

## 4. Discussion

The present experiment demonstrated that an ethanol extract of* F. mume *rescued scopolamine-induced impairment of the acquisition and retention of spatial memory, based on improved results in the Morris water maze task and reduced induction of hippocampal AChE. In addition,* F. mume *treatment increased ChAT levels in the hippocampus. Specifically, at all concentrations tested,* F. mume* was effective in rescuing the cognition deficits induced by scopolamine. Furthermore, the positive control, donepezil, was also effective, which is consistent with a previous report [27]. However, scopolamine-treated mice that also received donepezil showed no significant improvements in the second retention test (the second probe trial) compared to scopolamine-treated mice. This might be as a result of debilitating effects of donepezil administrated over long periods of time. Donepezil is high risk for accumulation due to its high protein binding and has a long half-life, which might cause serious adverse events in animals [28].

*F. mume* is known for the fruit* of Prunus mume*, which belongs to Rosaceae family [3]. It contains many chemical compounds, such as 4-*O*-caffeoylquinic acid methyl ester, prunasin, 5-*O*-caffeoylquinic acid methyl ester, benzyl-*O*-*β*-d-glucopyranoside, and liquiritigenin-7-*O*-*β*-d-glucopyranoside [25]. A number of studies have shown that* F. mume* exerts antibacterial effects, antioxidants, and antiosteoporosis activities [29, 30]. Most studies reporting the effectiveness of* F. mume* have used its ethanol, methanol, and water extract [3, 24, 29, 30]. But the recent study demonstrating ameliorative effects of* F. mume *on glucose intolerance and fat accumulation reported that the ethanol extract increased glucose uptake in C2C12 myotubes more than its water extract [24]. Hence, the present study used the ethanol extract of* F. mume* and examined its effects on scopolamine-induced memory impairment.

As reported previously [23], in the present experiment, scopolamine increased AChE levels in the hippocampus, which was reversed by treatment with* F. mume* (200 mg/kg) or donepezil. Although there were no differences in hippocampal ChAT expression between the vehicle-control and scopolamine-treated mice, increased expression levels of ChAT were observed in the hippocampus of scopolamine-treated mice that also received* F. mume*, at all concentrations. Donepezil treatment also increased the hippocampal expression levels of ChAT in scopolamine-treated mice.

We used scopolamine to investigate the effects of* F. mume* on the cholinergic neurons given that it is reported to induce ACh depletion in the mouse hippocampus [22]. Reduced ACh may be responsible, in part, for the cognitive deficits in animals after scopolamine treatment [31, 32]. The mechanism of action of the effects of* F. mume* on cognition, therefore, would be via the cholinergic septohippocampal system, as was evidenced by decreased AChE levels and increased ChAT in the hippocampus of scopolamine-treated mice that also received* F. mume*. However, further study is required to reveal the other mechanisms and brain structures responsible for the effect of* F. mume* (e.g., cholinergic system from the BFCNs to the cortex).

Compared to their age-matched littermates, transgenic mice with the human ChAT gene exhibited improvements in cognition during the aging process [33]. Furthermore, exogenous administration of ChAT attenuates cognitive impairments in aged mice [34]. Natural products, including coumarins, flavonoids, and stilbenes, are AChE inhibitors and represent a source of new lead compounds for the development of AD treatments [35]. In this context, the present finding that* F. mume* decreased AChE activity and increased expression levels of ChAT indicates that it might also fit this purpose.

Recently we reported the effectiveness of an* F. mume* extract in treating VaD [3]. The effectiveness of* F. mume *was examined in rats with chronic cerebral hypoperfusion.* F. mume* treatment ameliorated the cognitive deficits in rats with chronic BCCAo. In addition, the molecular mechanisms of this effect of* F. mume* were examined in this study.* F. mume* reduced microglial activation, phosphorylation of extracellular-signal-regulated kinase (ERK), and nuclear factor kappa B (NF-*κ*B) activity in the chronic BCCAo hippocampus. Therefore, this report indicates that* F. mume* might be a potent anti-inflammatory treatment for CNS diseases associated with neuroinflammation. However, because cholinergic markers in the hippocampus are altered in chronic BCCAo rats [7–9], given the results of the present experiment, there is a possibility that* F. mume *might rescue cognitive impairment in chronic BCCAo rats via an action mediated by the cholinergic neurons.

## 5. Conclusions

We showed that* F. mume* ameliorated scopolamine-induced cognitive deficits in mice. Considering that* F. mume* treatment reversed the scopolamine-induced increases in hippocampal AChE level and increased hippocampal ChAT levels, the present finding is the first evidence that* F. mume* enhances the cholinergic system, thereby improving cognitive function. Therefore, these results suggest* F. mume* as a promising natural product for the prevention of memory disorders and AD.
